# Mitochondrial dynamics in the neonatal brain – a potential target following injury?

**DOI:** 10.1042/BSR20211696

**Published:** 2022-03-23

**Authors:** Adam Jones, Claire Thornton

**Affiliations:** 1 Department of Biomedical Engineering, School of Biomedical Engineering and Imaging Sciences, King’s College London, London SE1 7EH, U.K.; 2 Department of Comparative Biomedical Sciences, Royal Veterinary College, London NW1 0TU, U.K.

**Keywords:** cell death, hypoxia, ischaemia-reperfusion injury, mitochondrial dynamics, neonatal

## Abstract

The impact of birth asphyxia and its sequelae, hypoxic–ischaemic (HI) brain injury, is long-lasting and significant, both for the infant and for their family. Treatment options are limited to therapeutic hypothermia, which is not universally successful and is unavailable in low resource settings. The energy deficits that accompany neuronal death following interruption of blood flow to the brain implicate mitochondrial dysfunction. Such HI insults trigger mitochondrial outer membrane permeabilisation leading to release of pro-apoptotic proteins into the cytosol and cell death. More recently, key players in mitochondrial fission and fusion have been identified as targets following HI brain injury. This review aims to provide an introduction to the molecular players and pathways driving mitochondrial dynamics, the regulation of these pathways and how they are altered following HI insult. Finally, we review progress on repurposing or repositioning drugs already approved for other indications, which may target mitochondrial dynamics and provide promising avenues for intervention following brain injury. Such repurposing may provide a mechanism to fast-track, low-cost treatment options to the clinic.

## Introduction

The cellular energy required by the developing brain around the time of birth and during early life is substantial, emphatically illustrated by the rapid increase from 27 to 80% of its adult weight within the first 2 years of life [1]. It is unsurprising, therefore that efficiently functioning mitochondria coupled with a ready supply of oxygen are pivotal to meet the ATP demands of a rapidly expanding brain. However, mitochondria not only live up to their ‘powerhouse’ reputation, they are also hubs for immune response, calcium buffering as well as regulating reactive oxygen species (ROS) production and triggering programmed cell death [2–4]. This expanded repertoire of functions ascribed to mitochondria has emerged in part due to significant advances in our understanding of its molecular regulation. It is now clear that in addition to the roles mentioned above, mitochondria can adapt morphologically, dictated by the presence or absence of environmental stress, and that such dynamic adaptations are tailored to specific mitochondrial functions [5,6]. Mitochondrial dynamics, the process of balanced cycling through fission and fusion, are essential for maintaining cellular health and dysfunctional dynamics have been implicated in almost all pathological conditions with any element of bioenergetics compromise [6]. Focussing on the brain, perturbations in mitochondrial dynamics have been identified in numerous neurodegenerative conditions (e.g. Alzheimer’s disease, Parkinson’s disease, Amyotrophic Lateral Sclerosis) as well as stroke, traumatic brain injury and neurodevelopmental conditions such as Autism Spectrum Disorder [7–11]. In this review, we introduce the molecular players involved in regulating mitochondrial dynamics, and then describe the effect of hypoxic–ischaemic (HI) injury, particularly after birth asphyxia, on these processes. Finally, we evaluate the potential of repurposing drugs in order to provide therapies to restore balanced mitochondrial fission and fusion, which may be considered for therapeutic intervention in neonatal hypoxic–ischaemic encephalopathy (HIE).

## Mitochondrial health is managed by interconnected mechanisms

**Figure 1:**
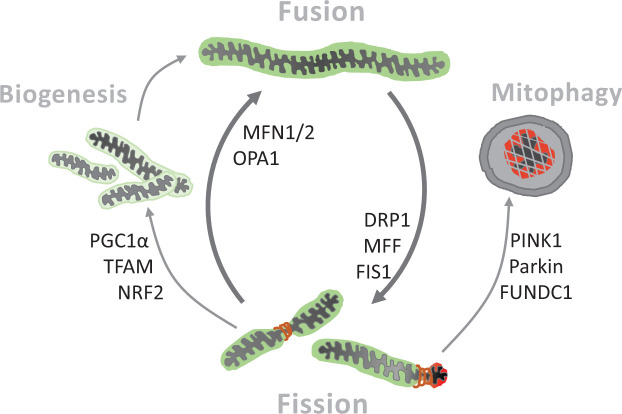
Mitochondria morphology is highly regulated Mitochondria cycle through fission and fusion with new mitochondria being generated through biogenesis and mitochondrial quality control managed by mitophagy. The GTPases OPA1 and mitofusins (MFNs) 1 and 2 regulate IMM and OMM fusion respectively. The GTPase DRP1 migrates from the cytosol to bind to one of a number of proteins (FIS1, MFF) located at the OMM to instigate fission. Midpoint fission generates small mitochondria for biogenesis and asymmetrical fission segregates damaged mitochondria for recycling via mitophagy. Abbreviations: IMM, inner mitochondrial membrane.

Mitochondrial function is governed by a series of regulated mechanisms that, in addition to fusion and fission (division [5]), include mitochondrial biogenesis [12] and mitophagy (Figure 1; [13]) as well as elegant machinery designed to transport the mitochondria around the microtubule network [14]. Mitochondrial fusion provides a means to share mitochondrial contents including mitochondrial DNA (mtDNA), proteins and lipids. As such, minor defects such as low-level mtDNA mutation or accumulating ROS can be ‘diluted out’ by fusion of the impaired mitochondrion into more complex mitochondrial network [15]. Mitochondrial fusion can also promote increased oxygen consumption, oxidative phosphorylation and therefore higher yields of ATP [16]. Mitochondrial fission is essential for quality control, as fragmentation of a large mitochondrion into smaller parts can isolate areas of damage for subsequent clearance. Biogenesis provides new mitochondria for the cellular pool, whereas mitophagy is the process of recycling dysfunctional mitochondria, for example with damaged mtDNA or with loss of mitochondrial membrane potential (∆Ψm; [17]).

## The molecular mediators of mitochondrial dynamics

As mitochondria are double membrane-bound organelles, mitochondrial fusion requires two separate events, one to fuse the inner mitochondrial membrane (IMM) and one for the outer mitochondrial membrane (OMM). Both are governed by large dynamin-related GTPases; mitofusin (MFN) 1 and MFN 2 are responsible for OMM fusion, with Optic Atrophy (OPA1) mediating IMM fusion [18]. Fission is driven by the GTPase Dynamin-related (Drp)1, normally cytosolic, which is recruited to the mitochondrion through binding to one of Mitochondrial Fission Factor (MFF), Mitochondrial Division (MiD) 49, MiD51 or Fission (Fis)1, located at the OMM [5].

### Mitochondrial fusion

OPA1 exists in long (L-OPA1) and short (S-OPA1) forms, both of which are required for OPA1 function [19–22]. OPA1 is imported into the mitochondria via an N-terminal mitochondrial targeting sequence, followed by mitochondrial processing peptidase (MPP) cleavage to generate L-OPA1 [23]. L-OPA1 localises to the mitochondrial intermembrane space, bound to the IMM by an N-terminal transmembrane (TM) domain [22–24]. L-OPA1 is then proteolytically cleaved to S-OPA by OMA1 and YME1L peptidases localised to the IMM; once cleaved, S-OPA is soluble. Co-operation between L-OPA1 and cardiolipin (a negatively charged lipid abundant in the IMM) is sufficient for IMM fusion although S-OPA1 can facilitate this process further [25]. IMM fusion is therefore dependent on OMA1 and YME1L activities to ensure the correct ratio of L-OPA and S-OPA; excessive cleavage of L-OPA1 will allow unopposed mitochondrial fission. OPA1 null MEFs results in a fragmented mitochondrial network due to fusion incompetence [21]. Complete ablation of OPA1 is embryonic lethal in mice, whereas overexpression is also toxic; however, mild overexpression is compatible with life and has shown OPA1 to be protective in response to cell death stimuli, along with restoration of ATP production and the morphology of mitochondrial networks [20,26–28].

MFN1 and MFN2 share 77% amino acid sequence similarity and are required for OMM fusion as they are capable of tethering neighbouring mitochondria, allowing GTP-dependent fusion to take place [29]. The activity of MFNs is regulated by post-translational modification, including ubiquitination and phosphorylation [30,31]. Phosphorylation of MFNs by PTEN-induced serine/threonine kinase (PINK)1 can promote subsequent ubiquitination by Parkin, leading to mitophagy and degradation [32,33]; degradation is also observed following ubiquitination by MARCH5 leading to mitochondrial fission [34]. ERK1/2 phosphorylates MFN1 leading to increased mitochondrial fission [35] although *in vivo* confirmation of this regulation remains [36]. MFN1 and MFN2 have tissue-specific expression and differences in GTPase activities [37,38]. The brain showed lower mRNA expression for both MFNs compared with heart, liver or skeletal muscle with MFN1 more highly expressed than MFN2 [38]. Purified recombinant MFN1 GTPase activity was approximately eight-fold higher than MFN2, whereas MFN2 has a higher affinity for binding GTP [37]. These differences may reflect evidence suggesting that MFN1 drives the initial tethering of the mitochondrial membranes together, whereas MFN2 may act in the tethering of the ER to the mitochondria [39]. Indeed although there is redundancy, genetic studies suggest individual roles for MFN1 and MFN2 during development [40]. However, assignment of such specific functions between MFNs is still being debated [41].

### Mitochondrial fission

The initiation of mitochondrial fission is reliant on organelle interaction and protein phosphorylation. Mitochondrial fission is dictated by the endoplasmic reticulum, which can wrap around the mitochondrion at the chosen site and initiate constriction [42]. DRP1 then translocates from the cytosol, binds to OMM protein partners to spiral around the mitochondrion, constricting and finally severing both mitochondrial membranes, powered by GTP hydrolysis [15,19,43–45]. DRP1 is regulated by multiple post-translational modifications, including phosphorylation at S616 by ERK, and S637 by Protein Kinase A (PKA) or AMP-activated protein kinase (AMPK [46,47]). Phosphorylation of DRP1 at S616 promotes its translocation to the mitochondria, and observations of DRP1-pS616 correlate with mitochondrial fragmentation [48–50]. DRP1 phosphorylation at S637 inhibits fission in part through attenuating DRP1 GTPase activity [51]. This inhibition is reversible as dephosphorylation by the calcium-dependent phosphatase calcineurin [52] and subsequent rephosphorylation at S616 results in fission [46,48–50,53]. Further regulation is provided by A-kinase anchoring protein (AKAP)1, which anchors PKA at the OMM in order to phosphorylate DRP1 at S637 [54]. Phosphoregulation of DRP1 at S637 promotes cell survival and has been identified as key factor in determining ischaemic sensitivity post-injury [46,48].

Unlike other members of the dynamin family, DRP1 does not possess a pleckstrin homology domain, which would normally facilitate phospholipid binding and membrane localisation. Therefore DRP1 recruitment to the mitochondrion relies on interaction with a number of binding partners already anchored to the OMM, including mitochondrial fission 1 protein (FIS1), MFF and mitochondrial dynamics protein (MiD)49/MIEF2 and MiD51/MIEF1 [46,55–57]. MFF is considered the major DRP1-binding partner, capable of promoting DRP1 GTPase activity, and loss of MFF results in significant mitochondrial fusion [58]. MFF is itself regulated by phosphorylation by AMPK that increases mitochondrial fission [59], and this may trigger an alternative pathway to mitophagy in response to cellular stress [60]. The role of FIS1 in DRP1-mediated mitochondrial fission is still being clarified as knockdown of FIS1 promotes fused mitochondrial morphology, but the interaction between DRP1 and FIS1 is weak [61]. A combined MFF/FIS1 knockout model determined an additive mitochondrial hyperfusion effect suggesting that FIS1 acts independently of MFF to promote fission [62]. One possible mechanism may be by inhibition of fusion rather than promoting fission; a recent study identified that FIS1 interacts strongly with pro-fusion OPA1 and MFNs, inhibiting their GTPase activity [61]. Alongside MFF, MiD49/51 bind and recruit DRP1 to the OMM, mediating DRP1 oligomerisation and its encircling of the mitochondrion. However, in contrast with MFF, MiD49/51 binding inhibits DRP1 GTPase activity [63]. Further uncertainty is added when considering that both MiD49/51 knockdown and overexpression studies yield a fusion phenotype [62]; a possible explanation may lie in the influence of cellular environment and the phosphorylation state of DRP1.

The fate of mitochondria following fission is based on mitochondrial ΔΨm. Healthy mitochondria are fed back into the cycle to be used as substrates for biogenesis, whereas those with perturbed ∆Ψm trigger the accumulation and dimerisation of PINK1 around the OMM [15,64]. On autophosphorylation, PINK1 recruits the E3 ubiquitin ligase Parkin, and ubiquitin, marking the mitochondrion for LC3B recruitment into an autophagosome and subsequent autolysosomal degradation, in a process termed mitophagy [65,66]. Of relevance to the HI brain was the identification of a PINK1/Parkin-independent pathway to mitophagy, regulated by hypoxia. Src-phosphorylated FUNDC1 is located at the OMM and becomes dephosphorylated during hypoxia which permits binding to LC3B and autolysosomal degradation [67]. Biogenesis from fissioned mitochondria ‘seeds’ relies on the replication of mtDNA, and subsequent transcription and translation of genes critical for mitochondrial function such as electron transport proteins [12]. Key regulators of this process are the transcription factors Tfam, NRF1/2 and PGC1α, this last being described as a master regulator of biogenesis [68]. A recent study has further identified mechanisms governing the fissioned mitochondrial fate, dependent on the position of the fission site [69]. Fission events taking place at a midpoint region of the mitochondrion were identified as DRP1–MFF-dependent and led to biogenesis, whereas those taking place at the periphery were governed by DRP1–FIS1 interactions. These FIS1-mediated fission events created smaller mitochondria which were subsequently targeted for mitophagy, minimising the mass of material to be recycled [69]. Peripheral fission events were found to follow a decrease in ∆Ψ_m_ and an increase in ROS and calcium signalling, promoting cell survival after stress stimuli [69]. However, mitochondrial biogenesis and mitophagic quality control are not the focus of this paper, and the reader is directed to excellent, comprehensive reviews [70–72].

## Energy metabolism fails in neonates after birth asphyxia

Birth asphyxia leading to HIE affects 1–2 in every 1000 infants in the United Kingdom and far more in low resource countries [73,74]. The consequences for babies severely affected are significant including long-lasting neurological impairments (cerebral palsy, epilepsy) and death. Clinical MRI studies report that HIE is characterised predominantly by evolving lesions in grey matter structures (thalamus, basal ganglia), cortical abnormalities and, to a lesser extent, associated white matter injury. Neurons are most vulnerable, with the extent of lesion size being strongly associated with the severity of long-term impairments [74].

**Figure 2:**
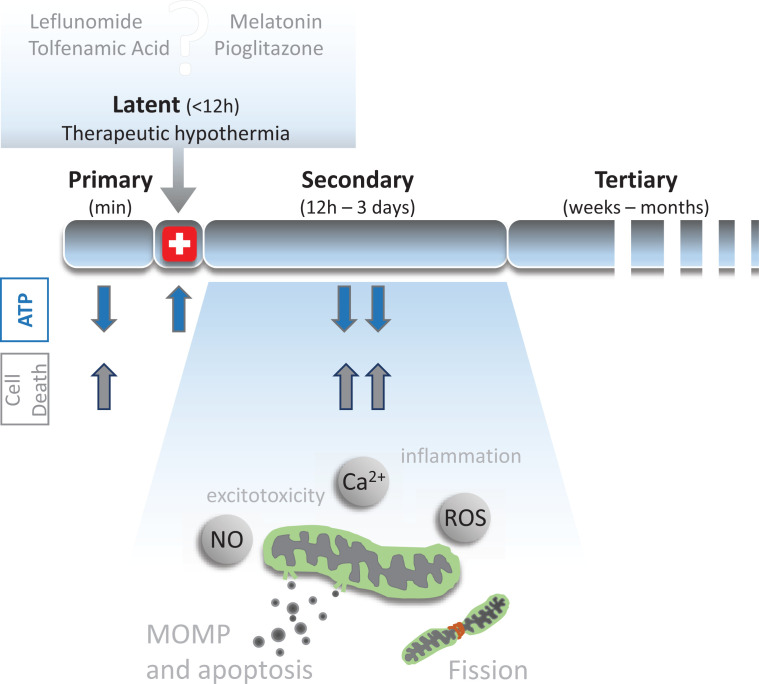
Secondary energy failure following birth asphyxia results in mitochondrial impairment The primary phase of injury lasts over the time of the asphyxia and results in initial ATP depletion and necrosis. A latent phase lasting from 1 to 12 h following injury occurs in which ATP levels recover to baseline and where therapeutic interventions are initiated. A secondary injury phase is characterised by multiple cellular processes impairing mitochondrial function leading to BAX-mediated mitochondrial outer membrane permeabilisation (MOMP), mitochondrial fission and culminates in cell death.

Brain injury following asphyxia occurs as a result of impaired neuronal energy metabolism and evolves with time, passing through distinct phases (Figure 2). The primary energy failure phase (30–60 min) encompasses the initial asphyxia insult depriving the tissue of oxygen and glucose, leading to immediate depletion of ATP, anaerobic respiration, excessive glutamate release and necrotic death [75,76]. Reperfusion results in latent phase 6–12 h after initial insult, characterised by a partial recovery from oxidative stress. However, a secondary energy failure phase occurs in which mitochondrial dysfunction, oxidative stress, neuroinflammation and programmed cell death are features, and which lasts up to 72 h [75,77–79]. Finally a tertiary (months) phase occurs in which chronic neuroinflammation persists and neurogenesis and myelination are impaired [80].

The latent phase is considered a therapeutic window for intervention as high-energy phosphate levels are recovered to baseline levels due to partial restoration of tissue oxygenation [75,81]. However, currently the only clinically available treatment is therapeutic hypothermia (cooling to 33–34°C for 72 h, following by a slow rewarming at 0.5°C per hour), initiated within 6 h of birth [82–86]. Therapeutic hypothermia acts to restore metabolic and cellular energy state homoeostasis and provides proof-of-principle that a therapy administered following the initial injury can be effective in preventing the long-lasting effects of HI. However, it has a limited success rate, highlighting the need for additional synergistic or stand alone therapy [84,86,87].

### Mitochondria permeabilise following neonatal HI injury

HI injury triggers an array of pathophysiological outcomes (Figure 2). Following the initial HI injury, a cascade of signalling events is initiated including calcium overload, inflammation, generation of ROS and excitotoxicity and culminating in activation of a variety of programmed cell death pathways [88–94]. Mitochondrial dysfunction is a key characteristic linking these diverse observations [95,96] and in animal models of neonatal HI, oxidative phosphorylation and calcium homoeostasis are impaired [97–99]. Indeed electron microscopy identified ultrastructural changes in mitochondrial cristae due to calcium influx following HI in neonatal rats [90]. In addition, in response to HI, the OMM becomes leaky due to the formation of activated Bax/Bak pores allowing passage of cytochrome *c* and apoptosis-inducing factor (AIF) from the mitochondrial intermembrane space (IMS) into the cytosol. Once released, AIF and cytochrome *c* initiate a cascade resulting in activation of caspases, degradation of DNA and ultimately apoptotic cell death [100,101]. As such, genetic and pharmacological inhibition of Bax is protective from HI in the immature brain [102,103].

### Mitochondrial dynamics are targeted in the brain following ischaemia–reperfusion injury

Imbalance of mitochondrial fission and fusion has been observed in a number of adult ischaemia–reperfusion (I/R) injuries including kidney [104] and heart [105], where mitochondrial fission is a driver of injury. Genetic or pharmacological inhibition of DRP1 or promotion of mitochondrial fusion results in increased cell survival and restoration of tissue function [106–108].

Similar observations have been made in brain in models of cerebral ischaemia where increased mitochondrial fission (or decreased fusion) is associated with cell death and development of injury [109]. *In vitro*, increased fission is observed following oxygen-glucose deprivation in hippocampal [110,111] and microglial [112] cell lines, neurons [79,113], astrocytes [114] and microglia [115]. Increased mitochondrial fission and/or pro-fission DRP1 phosphorylation is apparent in rodent models of middle cerebral artery occlusion (MCAO) and DRP1 inhibition results in preservation of tissue [110,116,117]. Mitochondrial fission is also promoted through influx of calcium, activating the phosphatase calcineurin and subsequently lifting the fission-inhibitory phosphorylation at DRP1-S637 [52]. Supporting the link between S637 dephosphorylation and exacerbation of injury, DRP1-S637 phosphorylation is reduced and the infarct volume increases following MCAO in mice lacking AKAP [46]. Long forms of OPA1 are also a target for cleavage by peptidases such as OMA1 following MCAO [28,118] and global ischaemia [111]. S-OPA1, together with pro-apoptotic cytochrome *c* have been found to be released into the cytosol, ultimately leading to cell death [119]. OPA1 is also targeted by ischaemic insult *in vitro*. L-OPA1 is cleaved to S-OPA in primary rodent neurons following OGD, correlating with mitochondrial fragmentation [79,119]. Interestingly, efforts to maintain OPA1 expression can resist this ischaemia-mediated bias towards fission [28,118] presumably overcoming mechanisms promoting pro-fission DRP1 phosphorylation.

In the neonatal brain, alterations in mitochondrial dynamics have been demonstrated *in vivo* using the Vannucci model of neonatal HI. Increased gene expression of MFN1 and FIS1 [120] and decreased expression of OPA1 [79] has been observed at 24 h post-injury; no changes have been observed at earlier time points [79,120]. Post-translational modification of OPA1 occurs soon after HI resulting in increased cleavage of L-OPA1 to S-OPA1 and remaining through 24 and 48 h post-injury [79,120]. Decreases in phosphorylated DRP1 (S637) are also reported at 24 h post-injury indicative of a shift towards fission, although FIS1 protein expression over this period remains unchanged despite the increases in gene expression [120]. Decreased mitochondrial size was observed in brain sections stained for the mitochondrial marker TOMM20 at 24 h following neonatal HI [121]. The authors also found that hypoxia alone triggers decreased mitochondrial size but HI induced a more profound change, which was sex-specific; fission was more prominent in male compared with female ipsilateral sections [121].

## Pharmacological regulators of mitochondrial dynamics – potential therapies?

There is an urgent need to identify new treatments for neonatal brain injury following birth asphyxia and a number of potential therapies are showing promise [122]. However, given the impact of impaired fission and fusion on multiple aspects of the injury, deriving therapeutic compounds targeting mitochondrial dynamics may address the current paucity of treatments. Although the majority of these are compounds still in preclinical development (e.g. echinacoside [123], Drpitor [124]), there are an increasing number of studies centred around repurposing (or repositioning) drugs already in use clinically, with advantages including lower cost, reduced development time and pre-existing safety profiles [125]. There are a number of repurposed therapies reported to alter mitochondrial respiration (e.g. Methylene Blue [126]), however, here we will focus on those currently emerging treatments (in clinical use for other indications) that exert at least part of their effects through modulation of mitochondrial dynamics.

### Leflunomide

Leflunomide is approved for rheumatoid arthritis treatment as an anti-inflammatory, and is being considered for repurposing as a pancreatic cancer therapy [127]. A recent high-throughput screen identified Leflunomide as a small molecule activator of mitochondrial fusion through a pathway dependent on MFN1/2. Biochemically, Leflunomide inhibits dihydroorotate dehydrogenase (DHODH), an IMM enzyme that catalyses *de novo* synthesis of pyrimidines. Depletion of pyrimidines results in increased expression of MFN1/2 and mitochondrial fusion and pretreatment with Leflunomide prevented doxorubicin-mediated apoptosis in MEFs [128,129]. Leflunomide was also found to oppose mitochondrial fission in triple-negative breast cancer cells [130]. Leflunomide pretreatment (10–40 mg/kg) has preserved mitochondrial Δψm, reduced apoptosis and provided antioxidant effects in adult rodent I/R models in the kidney[131,132], intestine [133] and liver [134]. However, Leflunomide has not been tested in neonatal disease models post-injury and the dosage used in the I/R studies was significantly higher than in use for rheumatoid arthritis (adults: 100 mg per day initially, then 10–20 mg per day).

### Pioglitazone

Pioglitazone (PGZ) is a member of the thiazolidinedione family used to reduce insulin resistance and regulate blood glucose, hence in clinical use for type 2 diabetes [135]. PGZ acts as a PPAR agonist, known to increased PGC1α activity and mitochondrial biogenesis [136]. However, more recently, PGZ was shown to protect the expression of fusion (OPA1, MFN1/2) and fission (DRP1) proteins in a rabbit model of diabetes-induced atrial remodelling [137]. PGZ had similar effects in trisomic fetal fibroblasts where OPA1 and MFN1 gene expression was restored and DRP1 inhibited [138]. Pretreatment with PGZ (20 nmol) resulted in a reduction in DRP1-pS616 in the hippocampus following transient global ischaemia, which correlated with a reduction in oxidative stress and cell death [139]. On postnatal day 5 rat model of neonatal inflammation-induced brain injury, (mimicking preterm neuroinflammation), PGZ (20 mg/kg) reduced white matter damage and restored mitochondrial function [140]. Although PGZ is not routinely used in paediatric patients, small studies have shown no adverse effects in children [141] and adolescents ([142], 60 mg daily, equivalent to approx. 2.4 mg/kg). However, neonatal HIE would only require treatment for a short period and higher doses may therefore be feasible.

### Melatonin

Although considered a dietary supplement and therefore not regulated in the United States, Melatonin is indicated for use in Europe and the United Kingdom for insomnia. Melatonin is a naturally occurring hormone that regulates circadian rhythm and coincidentally is currently in clinical trials for neonatal HI injury [143]. As well as its antioxidant properties, Melatonin is reported to protect OPA1-mediated mitochondrial fusion in models of myocardial I/R injury [144,145], vascular calcification [146], diabetic fatty kidneys [147] and MCAO [148] most commonly via activation of AMPK.

### Tolfenamic acid

Tolfenamic acid is an anti-inflammatory, cyclooxygenase-2 inhibitor, which is in clinical use for treatment of acute migraine [149] and is also being investigated for efficacy in neurodegenerative conditions such as Alzheimer’s disease [150] and Huntington’s disease [151]. However, it was recently identified in a yeast-based screen of FDA-approved small molecules to rescue loss-of-function mutations in OPA1 that lead to dominant optic atrophy [152]. In patient fibroblasts harbouring mutations in OPA1, Tolfenamic acid restored mitochondrial morphology, mildly enhanced ATP synthesis and decreased ROS production [152]. Interestingly, previous molecular analysis identified CDK5 inhibition as a target for tolfenamic acid [153]. As CDK5 phosphorylates Drp1, it is possible that tolfenamic acid inhibits DRP1-mediated fission rather than acting directly on OPA1.

## Summary

Brain injury in the neonatal brain occurring around the time of birth leads to life-long neurological impairment and there is a clear unmet need for additional therapies to supplement or replace the use of therapeutic hypothermia. Mitochondria in neurons are vulnerable to the pathological effects of HI, both in the neonatal and the adult brain and the insult can trigger intrinsic apoptosis (via Bax-mediated MOMP) as well as perturbations of mitochondrial dynamics. As the molecular players driving the balanced actions of mitochondrial fission and fusion become more defined, specific interventions targeting these pathways for therapeutic benefit become more realistic. Repurposing drugs already in clinical use may offer a rapid and cost-effective route through to new treatments for neonatal HIE.

## Open Access

Open access for this article was enabled by the participation of Royal Veterinary College in an all-inclusive *Read & Publish* agreement with Portland Press and the Biochemical Society under a transformative agreement with JISC.

## Abbreviations

AIF: apoptosis-inducing factor
AKAP: A-kinase anchoring protein
AMPK: AMP-activated protein kinase
Drp: dynamin-related protein
FIS1: Fission 1
HI: hypoxic–ischaemic
HIE: hypoxic–ischaemic encephalopathy
IMM: inner mitochondrial membrane
I/R: ischaemia–reperfusion
L-OPA1: long optic atrophy 1
MCAO: middle cerebral artery occlusion
MFF: mitochondrial fission factor
MFN: mitofusin
MiD: mitochondrial division
MPP: mitochondrial processing peptidase
mtDNA: mitochondrial DNA
OMM: outer mitochondrial membrane
OPA1: optic atrophy 1
PGZ: Pioglitazone
PINK: PTEN-induced serine/threonine kinase
PKA: Protein Kinase A
ROS: reactive oxygen species
S-OPA1: short optic atrophy 1
Δψm: membrane potential
